# Targeting CD40 enhances antibody- and CD8-mediated protection against respiratory syncytial virus infection

**DOI:** 10.1038/s41598-018-34999-z

**Published:** 2018-11-09

**Authors:** Abenaya Muralidharan, Marsha Russell, Louise Larocque, Caroline Gravel, Changgui Li, Wangxue Chen, Terry Cyr, Jessie R. Lavoie, Aaron Farnsworth, Michael Rosu-Myles, Lisheng Wang, Xuguang Li

**Affiliations:** 1 Centre for Biologics Evaluation, Biologics and Genetic Therapies Directorate, HPFB, Health Canada and WHO Collaborating Center for Standardization and Evaluation of Biologicals, Ottawa, ON Canada; 2 National Institute for Food and Drug Control and WHO Collaborating Center for Standardization and Evaluation of Biologicals, Beijing, China; 3 Department of Biochemistry, Microbiology and Immunology, Faculty of Medicine, University of Ottawa, Ottawa, ON Canada; 4 Human Therapeutics Portfolio, National Research Council of Canada, Ottawa, ON Canada

## Abstract

Respiratory Syncytial Virus (RSV) infects almost all children under the age of one and is the leading cause of hospitalization among infants. Despite several decades of research with dozens of candidate vaccines being vigorously evaluated in pre-clinical and clinical studies, there is no licensed vaccine available to date. Here, the RSV fusion protein (F) was fused with CD40 ligand and delivered by an adenoviral vector into BALB/c mice where the CD40 ligand serves two vital functions as a molecular adjuvant and an antigen-targeting molecule. In contrast to a formaldehyde-inactivated vaccine, the vectored vaccine effectively protected animals against RSV without inducing enhanced respiratory disease. This protection involved a robust induction of neutralizing antibodies and memory CD8 T cells, which were not observed in the inactivated vaccine group. Finally, the vectored vaccine was able to elicit long-lasting protection against RSV, one of the most challenging issues in RSV vaccine development. Further studies indicate that the long lasting protection elicited by the CD40 ligand targeted vaccine was mediated by increased levels of effector memory CD8 T cell 3 months post-vaccination.

## Introduction

Respiratory Syncytial Virus (RSV) causes severe disease in young children, elderly and immunocompromised patients^1–4^. It is the leading cause of hospitalization in infants^1,2,5,6^ with approximately 50% of children being infected in their first year of life^7,8^. In the 1960s, a clinical trial involving formaldehyde-inactivated RSV (FIRSV) resulted in hospitalization of 80% of the vaccinees and 2 deaths following subsequent RSV infection^9–12^. Similar to the symptoms observed in the trial participants, FIRSV has been shown to induce a Th2-biased immune response leading to pulmonary inflammation, airway obstruction and mucus hypersecretion in many animal models, which are now deemed as the hallmarks of vaccine-induced enhanced respiratory disease (ERD)^13–16^. Moreover, non-neutralizing antibodies induced by FIRSV have been implicated in ERD development^17–19^, while another major facet of immunity, subsets of CD4+ T cells, was implicated in mediating various parameters of FIRSV-induced ERD^20,21^. However, the contribution of memory CD8 T cells in providing protection against RSV re-infection remains to be fully understood in spite of their known importance in viral clearance^20,22,23^. Indeed, eliciting a robust memory CD8 T cell response is thought to be the key in developing a vaccine that can promote long-lived immunity against RSV^22,24^.

CD40 and its ligand (CD40L) are a critical part of the adaptive immune system. In the adaptive immune response, antigen-presenting cells (APCs) must first be activated by an antigen with high affinity to MHC class I and/or II molecules on its surface. Next, the interaction of a receptor and its ligand occurs as a costimulatory signal necessary to initiate and regulate the response. Lastly, the activated APCs, CD8+ and CD4+ T cells activate cytokine release to carry out effector functions^25–27^. Interactions between CD40 and CD40L occur during the costimulation step and profoundly enhance the humoral and cell-mediated responses in addition to activating the APCs^28–30^.

CD40, part of the TNF receptor superfamily, is constitutively expressed on all APCs, activated CD4 T cells, CD8 T cells, fibroblasts, endothelial and epithelial cells^28–30^. CD40L, which is part of the TNF superfamily, is transiently expressed on activated CD4 T cells^28^ and may also be expressed on activated B cells, some dendritic cell subsets, platelets and smooth muscle cells^30^. Interactions between CD40 and CD40L have a considerable effect on promoting expansion and survival of APCs, T cells and B cells^29^. Moreover, CD40-CD40L is a crucial signal in stimulating CD4 T cells and in the process of direct or indirect priming of cytotoxic T lymphocytes by dendritic cells^28^. In B cells, engagement of the CD40 receptor improves antibody production, isotype switching, germinal center (GC) formation, and memory B cell maturation in addition to enhancing antigen presentation to T cells. Specifically, GC B cells undergo apoptosis after constant B cell receptor stimulation but T cell signals such as CD40L prevent this from happening, leading to longer antibody production^28,29,31^.

Previously, studies have shed light on the profound impact of targeting CD40 during RSV immunization using an anti-CD40 antibody or CD40L^32–34^. Nevertheless, separate administrations of the RSV antigen and CD40 targeting molecule were done and detailed mechanism of the immune responses, specifically cell-mediated responses, remain to be fully understood.

In this study, our goal was to develop and evaluate a vaccine expressing one protein consisting of both the RSV fusion (F) protein and CD40L. To the best of our knowledge, this is the first report of CD40L being used not only as a molecular adjuvant to enhance RSV F-induced host immunity, but also as an antigen-targeting molecule. Compared with FIRSV vaccine, the targeted vaccine induced higher levels of neutralizing antibodies while no ERD pathology was observed in the lungs. Further mechanistic studies indicate that the protection was dependent on CD8 but not CD4 T cells. Importantly, our study also demonstrated for the first time that it is feasible to induce CD8 T cell-mediated long-lasting protection through CD40-targeting immunization.

## Results

### Recombinant adenovirus construction and *in vitro* protein expression

**Figure 1:**
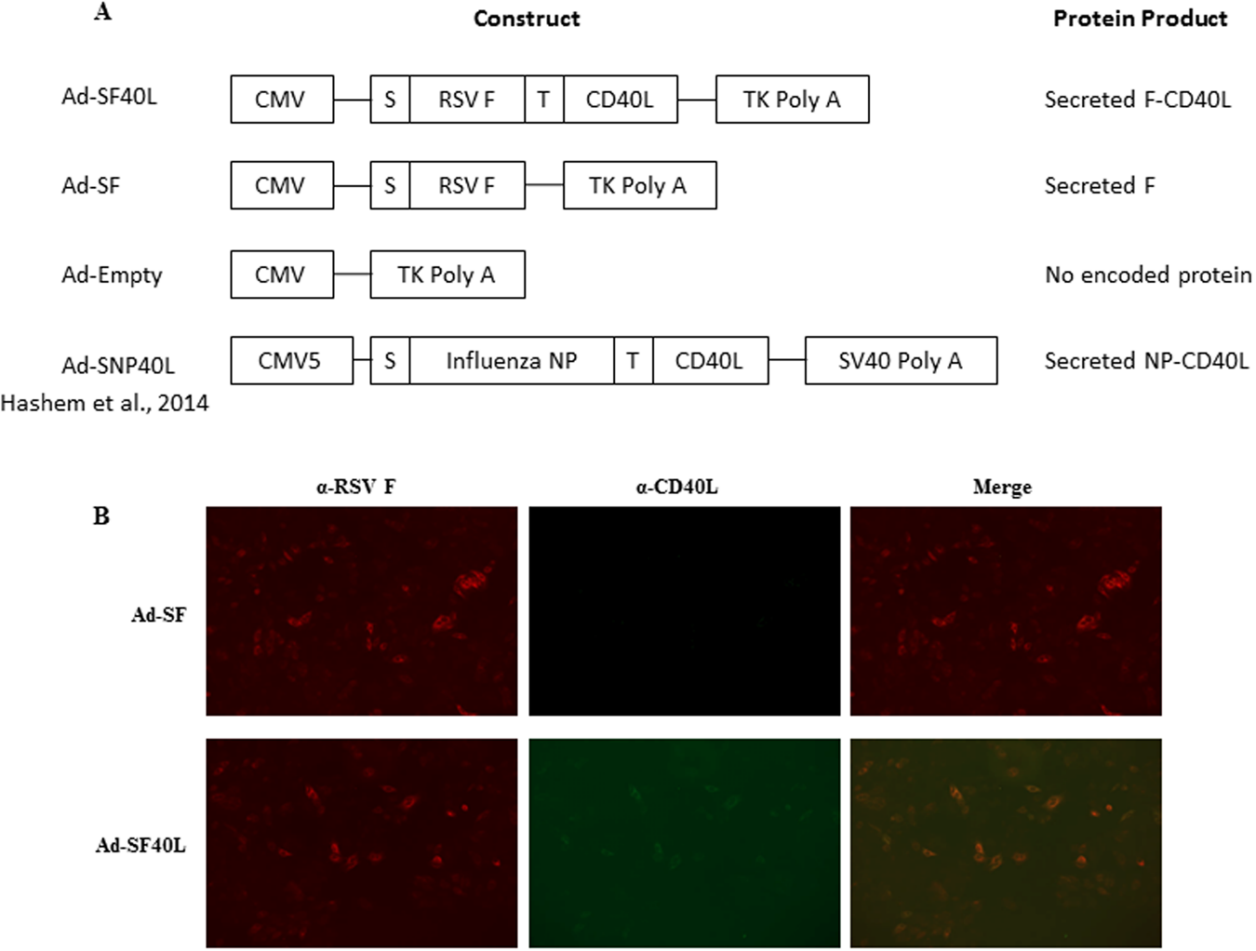
Non-replicating mouse codon-optimized recombinant adenovirus vaccines construction and *in vitro* protein expression. (**A**) Schematic representation of the Ad constructs. Both Ad-SF and Ad-SF40L express the full length RSV F protein preceded by a secretion signal, S. Ad-SF40L also encodes the full length mouse CD40 ligand, CD40L, following a trimerization motif, T. Ad-Empty does not encode for RSV F or CD40L whereas Ad-SNP40L encodes the influenza A nucleoprotein (NP) followed by CD40L as previously described. (**B**) Representative images at 10x magnification of *in vitro* protein expression following an immunofluorescence assay with a rabbit RSV F antibody along with Alexa Fluor 594-conjugated anti-rabbit IgG and FITC-conjugated anti-mouse CD40L. A merge of the two fluorochromes show the co-expression of RSV F and CD40L. Data are representative of two experiments.

Our aim was to determine if using CD40L as a molecular adjuvant during immunization can improve effective protection of mice from RSV infection. To that end, recombinant replication-deficient adenovirus expressing the full length RSV F protein, the most immunogenic of all RSV surface proteins^7^, fused to mouse CD40L were generated (Ad-SF40L) (Fig. 1A). A secretion signal and a trimerization motif were added to help increase antigen load, and to maintain the expressed CD40L in its functional trimeric form, respectively. To deduce the contribution of the CD40L to the observed protection, adenovirus expressing RSV F but not CD40L was also generated (Ad-SF). An empty adenovirus control (Ad-Empty) and antigen-specificity control expressing influenza nucleoprotein fused to CD40L (Ad-SNP40L)^35^ were also added to ensure that the protection detected was in fact due to the presence of both RSV F and CD40L.

Following the recombinant adenovirus construction, protein expression was tested *in vitro*. Immunofluorescence of HeLa cells infected with the adenoviruses confirmed the expression of RSV F protein in both Ad-SF and Ad-SF40L infected cells, whereas the expression of CD40L was only seen with Ad-SF40L (Fig. 1B). An image merging the fluorochromes attached to each antibody shows the co-expression of RSV F protein and CD40L by Ad-SF40L. Ad-Empty did not induce expression of either protein, as expected (data not shown).

### Immunization with Ad-SF40L augments RSV clearance without ERD in BALB/c mice

**Figure 2:**
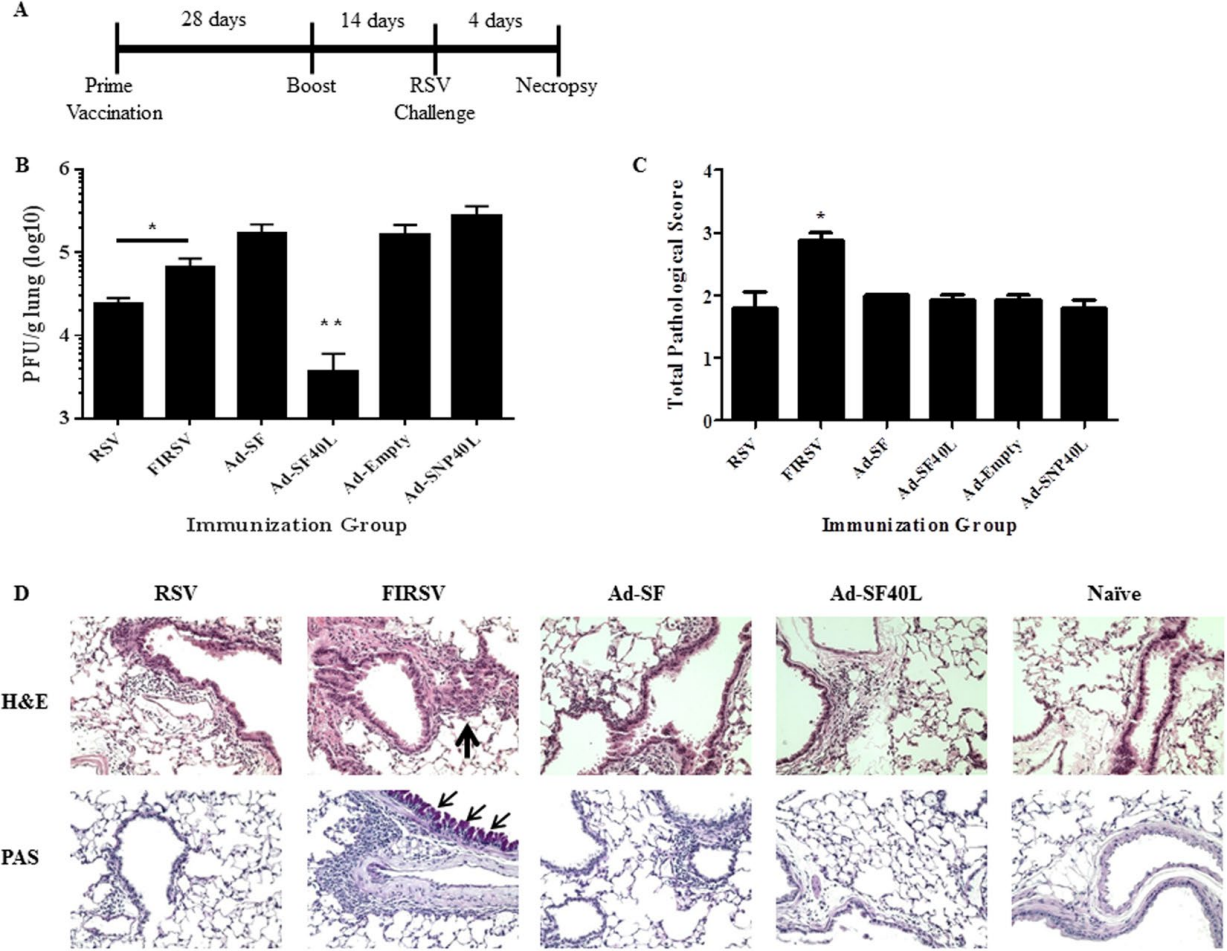
Immunization with Ad-SF40L augments RSV clearance without ERD in BALB/c mice. (**A**) Schematic diagram of the immunization, RSV challenge and necropsy timeline. (**B**) Lung viral titer determined using plaque assay 4 days post challenge. (**C**) Pathological scoring of lung tissue. Perivascular leukocyte infiltration and mucus were scored using H&E and PAS stained slides, respectively, 4 days following RSV challenge. An average of the two scores is shown. (**D**) Representative images of H&E and PAS stained immunized BALB/c mouse lungs post challenge at 40x magnification. The arrows point to the extensive cell infiltration in the H&E stained lungs and mucus-positive cells in the PAS stained lungs. Neutral mucins in airway epithelial cells are red when stained with a PAS stain. Data shown is mean ± SEM representative of 2 independent experiments; n = 4 per group in each experiment; *p < 0.05, **p < 0.01 (one-way ANOVA with Bonferroni posttest). FIRSV: Formaldehyde-inactivated RSV.

The adenoviruses produced were then tested in BALB/c mice for their ability of inducing protection against RSV. RSV and FIRSV groups were added to serve as controls for outcomes of a secondary infection and vaccine-induced ERD, respectively. All vectored vaccines and live RSV-A2 were immunized intranasally while FIRSV was injected intramuscularly. A prime-boost regimen was followed for all vaccines tested. Boost immunization was given 28 days following the prime and all mice were challenged with live RSV-A2 intranasally 14 days after the boost. Necropsy for tissue collection was conducted 4 days post challenge (Fig. 2A).

Ad-SF40L immunization resulted in significantly higher lung viral clearance than all other immunization groups (p = 0.0045) with an almost complete lung RSV clearance (Fig. 2B). Most importantly, these mice did not display any signs of ERD, demonstrating a safe, non-toxic protection (Fig. 2C,D). Clearly, the ERD pathology is only associated with FI-RSV vaccination. It is of note that even though Ad-SF immunization did not induce ERD, it failed to effectively clear the virus, similar to the two negative controls, i.e. Ad-Empty and Ad-SNP40L (influenza A NP construct control). These results collectively demonstrated the important role of CD40L in promoting RSV-specific immunity and robust viral clearance. Moreover, RSV immunization also resulted in better viral clearance than FIRSV immunization (p = 0.044) (Fig. 2B).

Lungs were H&E and PAS stained for histopathological analysis of interstitial disease, edema, perivascular aggregates of leukocytes and mucus. Mice from all immunization groups except FIRSV showed minimal perivascular cell infiltration and mucus levels whereas FIRSV immunized mice exhibited severe ERD displaying significantly higher cellularity and epithelial mucinous hyperplasia with luminal mucus accumulation in airways (p = 0.016) (Fig. 2C,D and Supplementary Fig. 1).

### Ad-SF40L induces high levels of neutralizing antibodies with the absence of a Th2-bias

Antibody-mediated immune responses are vital for robust protection against RSV^18^. The Th2-skewed nature of FIRSV-induced protection, especially in mice, has been well established^18,36^. This skew accompanied by the low levels of neutralizing antibodies detected post challenge has been shown in many studies as the major drawback associated with FIRSV-elicited immune responses^20,36,37^.

**Figure 3:**
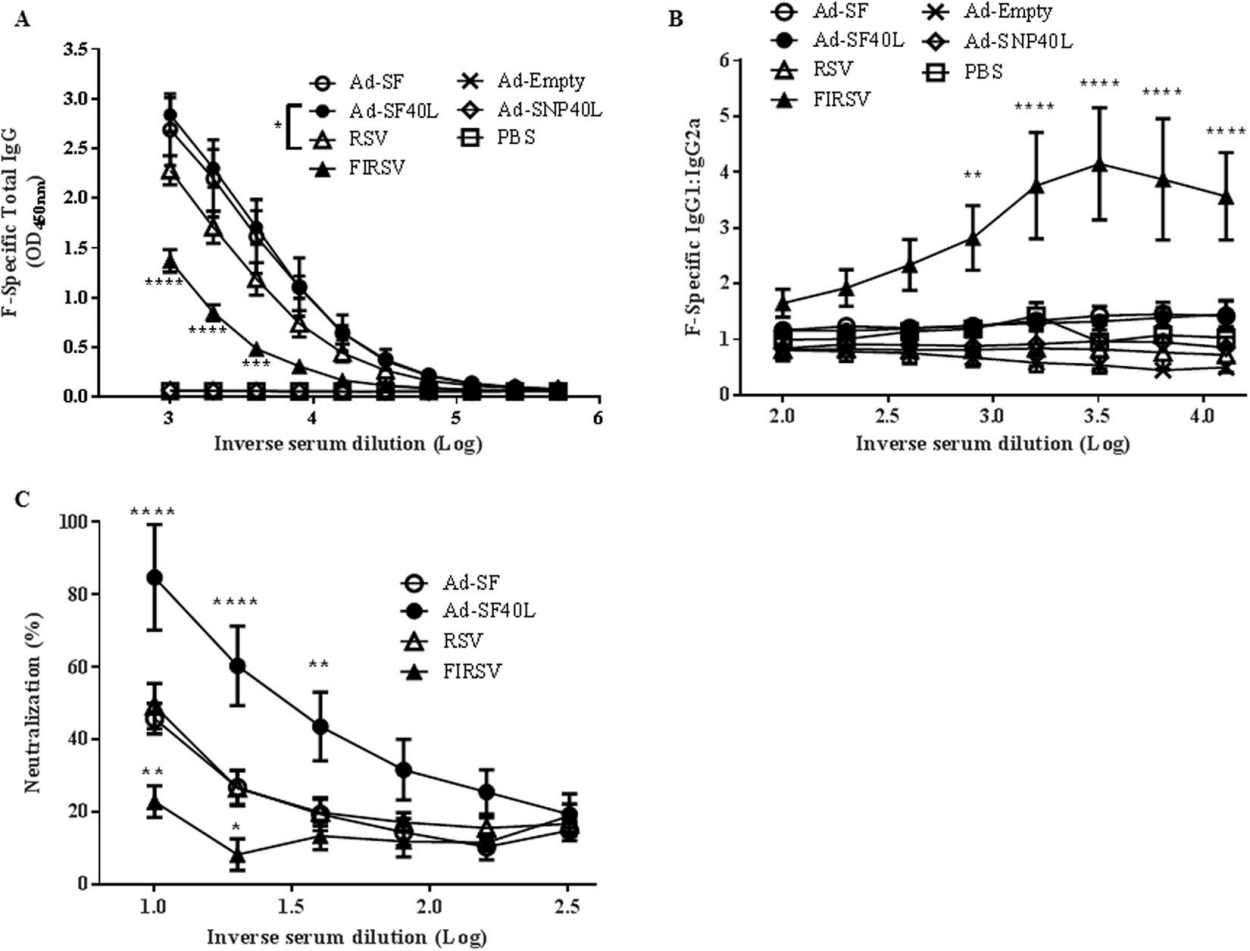
Ad-SF40L induces high levels of neutralizing antibodies. (**A**) F-specific total IgG in serum of primed/boosted mice 4 days post-challenge determined using ELISA (n = 5). The * in the legend indicates a significant difference in F-specific total IgG between Ad-SF40L and RSV at dilutions 1/1000, 1/2000 and 1/4000. (**B**) F-specific IgG1/IgG2a ratio in mice serum to show the Th2 or Th1 nature of the immune response (n = 5). (**C**) RSV neutralizing ability of the mice serum collected 4 days post-challenge (n = 8). Data shown is mean ± SEM representative of 2 independent experiments; *p < 0.05, **p < 0.01, ***p < 0.001, ****p < 0.0001 (two-way ANOVA with Bonferroni posttest).

In this study, we first confirmed these aspects of FIRSV-induced antibody responses where FIRSV-immunized mice had lower levels of RSV F protein-specific IgG than RSV, Ad-SF and Ad-SF40L immunized mice post challenge and highest Th2 subtypic profile, i.e. higher IgG1 to IgG2a ratio (Fig. 3A,B). Furthermore, the antibodies in FIRSV-immunized mice had very low RSV neutralizing abilities, as expected (Fig. 3C). Importantly, Ad-SF40L immunization induced high levels of F-specific IgG in the serum, even higher than live RSV immunization, without a Th2-bias. As there is no difference between Ad-SF and Ad-SF40L in terms of antigen specific IgG and IgG subtype, CD40L does not have a significant effect in this regard. However, the addition of CD40L during immunization contributes to a significant increase in neutralizing antibodies (p < 0.01) against RSV (Fig. 3C). Overall, these data indicate that while Ad-SF and RSV immunization gave rise to similar levels of neutralizing antibodies, Ad-SF40L induced the highest levels of neutralizing antibody among the vaccines tested.

### Increase in effector/effector memory CD8 T cells (TEM) following RSV challenge contributes to Ad-SF40L-induced protection

CD8 T cells play a critical role during infections and are sufficient to clear RSV^38,39^. Over time following infection or vaccination, as the antigen load decreases, the population of mature CD8 T cells contracts to form a stable memory CD8 T cell pool whose functional activities evolve with time^40^. As is the role of immune memory, RSV-specific CD8 T cells expand in magnitude and effector functions following infection^41^. However, FIRSV has been shown to elicit no memory CD8 T cell response in mice adding another facet to its ineffectiveness^42,43^.

**Figure 4:**
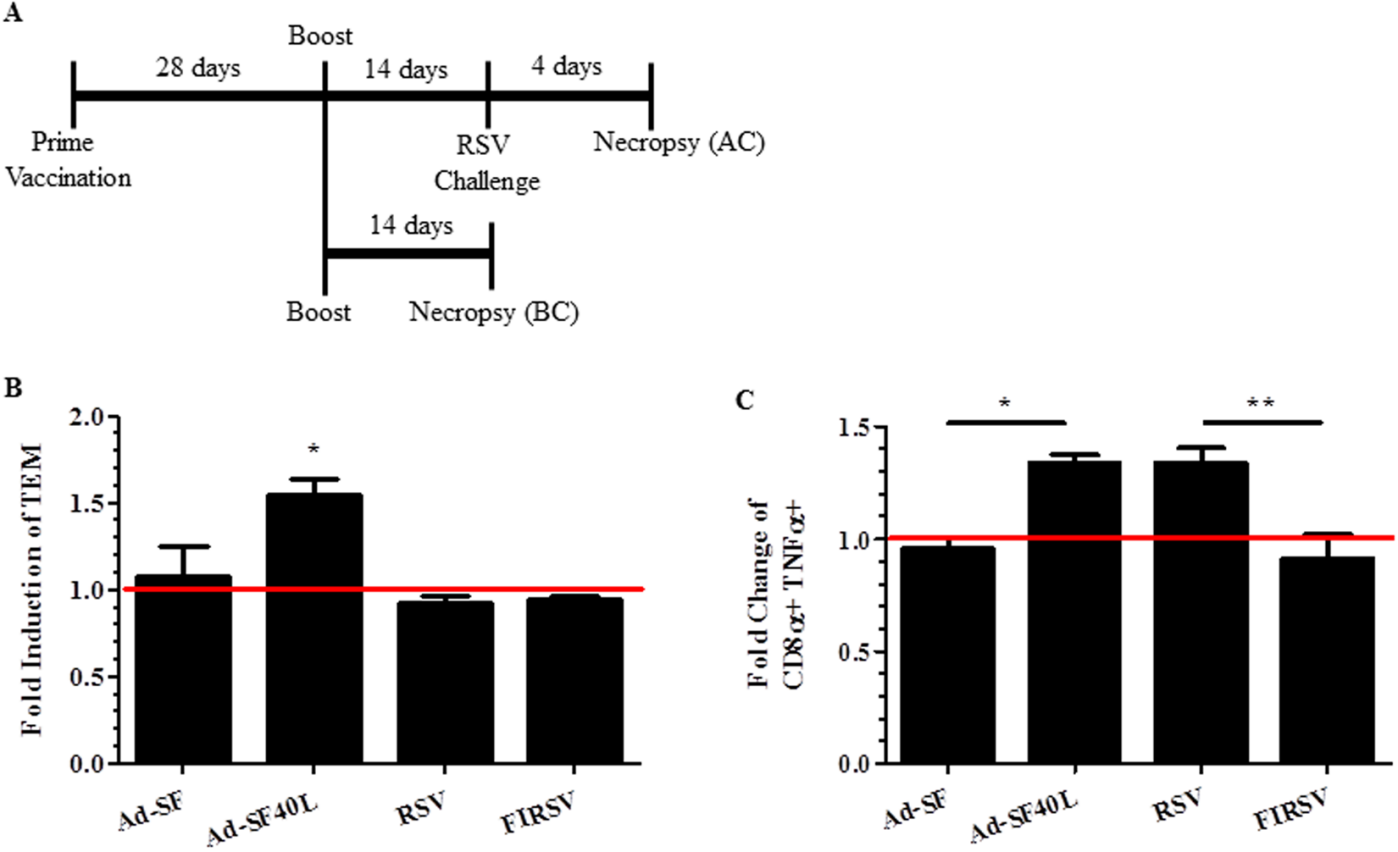
Increase in CD8 T cell effector phenotype and function following challenge is unique to Ad-SF40L immunization. (**A**) Schematic diagram of the animal study with BALB/c mice. Mice were necropsied for tissue collection before or after challenge. (**B**) Flow cytometry was used to determine the TEM population (CD3+ CD8a+ CD44+ CD62L− CCR7−) in the spleen. Induction of the TEM population as a result of the RSV challenge is shown as a ratio (TEM after challenge/TEM before challenge). A ratio of 1 indicates no change in the TEM population after challenge. (**C**) Intracellular cytokine staining was done following 4-hour *ex-vivo* stimulation with F85–93 peptide to analyze the number of CD8α+ TNF-α+ cells in the spleen using flow cytometry. Fold change of this population as a result of the challenge is shown (after challenge/before challenge). Data shown is mean ± SEM representative of 2 independent experiments; n = 4 per group; *p < 0.05, **p < 0.01 (one-way ANOVA with Bonferroni posttest).

To determine memory CD8 T cell-mediated immunity induced by Ad-SF40L and further characterize the CD8 population induced by FIRSV, we evaluated the CD8 T cell phenotype and functional changes that occur following RSV challenge. BALB/c mice were administered with the vaccines twice and euthanized either before or 4 days after challenge (Fig. 4A). Spleens were analyzed using flow cytometry for markers distinguishing various CD8 T cell phenotypes (Supplementary Fig. 2). The population of effector/effector memory CD8 T cells (TEM) after challenge significantly increased in mice immunized with Ad-SF40L (p < 0.05) compared to before challenge; no change was observed in Ad-SF, RSV and FIRSV immunization group post RSV challenge (Fig. 4B). Moreover, an increase in F-specific TNF-α producing CD8+ cells was only observed following challenge in Ad-SF40L and RSV immunized mice (Fig. 4C). The lack of increase in TEMs and TNF-α post-challenge in mice immunized with FIRSV may point to a defect in T cell memory maturation in FIRSV, which also occurs with Ad-SF, abrogating effective protection.

Following RSV infection, antigen-specific CD8 T cells exhibit an activated phenotype and gain effector functions^41^ corroborating the common theory that inflammatory responses increase with antigen encounters^40^. Notably, in humans, an increase in TEM population can be seen following resolution of infection^44^. Here, we found, unlike the other vaccination groups, Ad-SF40L resulted in complete viral clearance 4 days post-challenge (Fig. 2B), indicating that the resolution of infection was accompanied by an increase in TEMs. Additionally, following challenge, Ad-SF40L also increased expression of TNF-α, a potent inflammatory cytokine known to mediate RSV clearance^45,46^, confirming the presence of activated effector cells during the time of RSV resolution.

### CD40L enhances antibody-induced protection but not CD4 T cell-induced response

**Figure 5:**
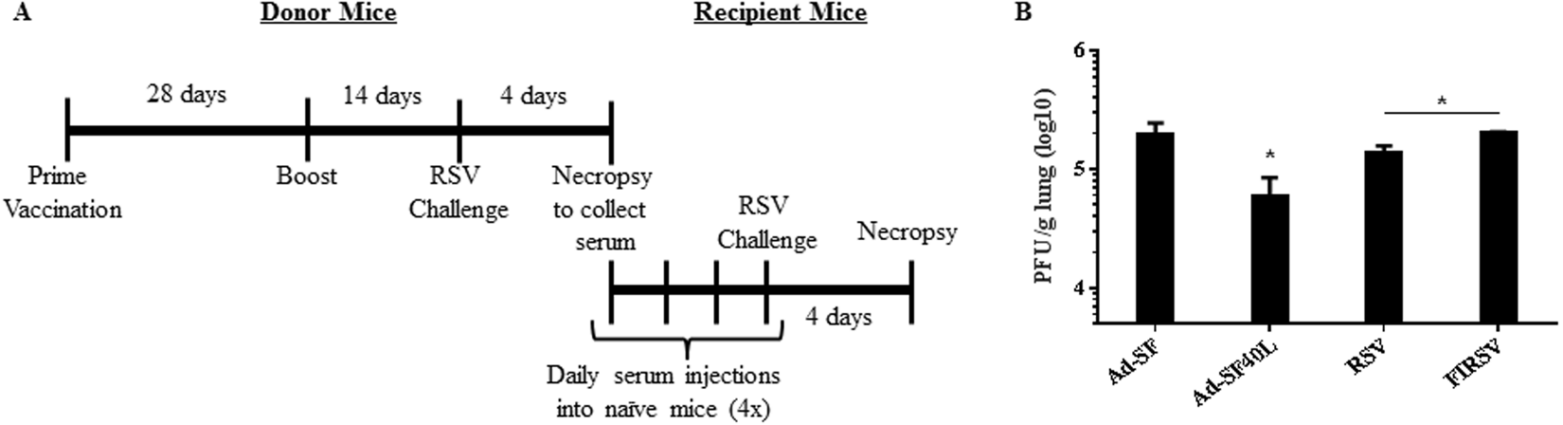
CD40L enhances antibody-induced protection. (**A**) Schematic diagram of the animal study timeline. Passive serum transfer was done from immunized and challenged donor mice into naïve recipient mice. (**B**) Four days post-challenge, lungs from the recipient mice were collected for viral titer determination using plaque assay. Serum from Ad-SF40L immunized mice resulted in the highest lung viral clearance in recipients. Data shown is mean ± SEM representative of 2 independent experiments; n = 4 per group; *p < 0.05 (one-way ANOVA with Bonferroni posttest).

Next we investigated the magnitude of protection afforded by antibodies derived from Ad-SF40L immunized mice in naïve mice. Figure 5A outlines the timeline followed for passive serum transfer where serum from immunized and challenged mice was transferred into naïve recipient mice that were subsequently challenged. As shown in Fig. 5B, four days post-challenge, lung viral titer was the lowest in mice that received serum from Ad-SF40L immunized donors compared to other groups, confirming the importance of the protective antibodies present in Ad-SF40L immunized donors (Fig. 3C). Since FIRSV immunization induced low F-specific IgGs that did not possess sufficient neutralizing ability (Fig. 3A,C), serum transfer expectedly failed to result in marked viral clearance in recipients (Fig. 5B).

Next, we examined the ability of CD4 T cells derived from each immunization groups in protecting naïve mice against RSV (Supplementary Fig. 4). No difference between Ad-SF and Ad-SF40L was found in viral clearance following adoptive CD4 T cell transfer even though Ad-SF40L resulted in better viral clearance than RSV (p < 0.01) and FIRSV (p < 0.05) groups. These results suggest that the protection induced by Ad-SF40L is CD4 T cell-independent.

### Marked increase in CD8 T cell effector phenotype and function following Ad-SF40L immunization

**Figure 6:**
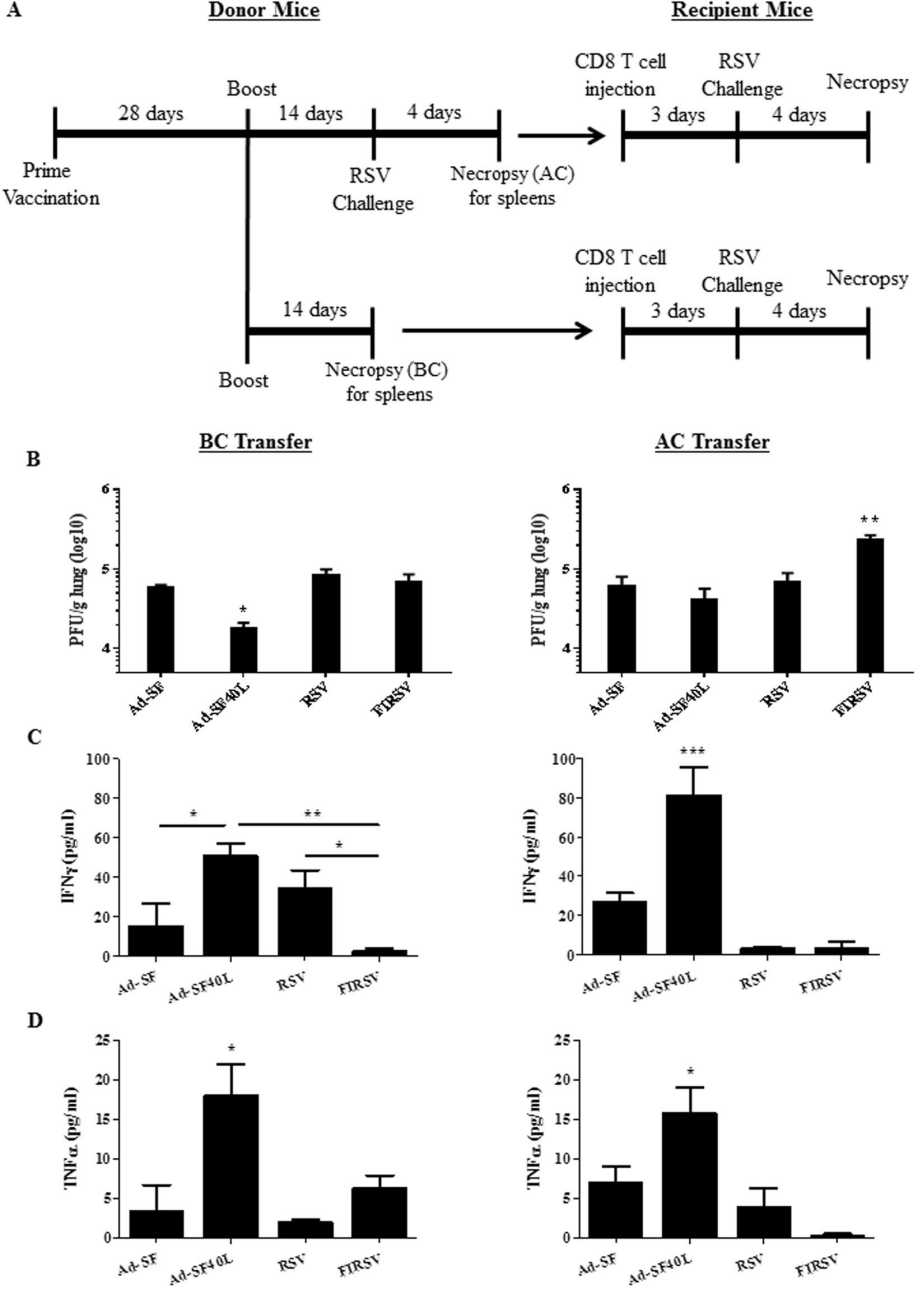
CD8 T cell effector function is altered following challenge of FIRSV-immunized mice unlike in Ad-SF40L immunization. (**A**) Schematic diagram of the animal study timeline. Adoptive CD8 T cell transfer was done from immunized donors either before (BC) or after (AC) challenge into naïve recipient mice. CD8 T cells were isolated from spleens of donor mice using a magnetic bead kit and checked for purity prior to transfer. (**B**) Four days post-challenge, lungs from the recipient mice from BC transfer (left) and AC transfer (right) were collected for viral titer determination using plaque assay. Ad-SF40L was compared to all groups (left) and FIRSV was compared to all groups (right). Comparisons between other groups showed no statistically significant differences. Secreted levels of IFN-γ (**C**) and TNF-α (**D**) were determined, using the Luminex system, in spleens of recipient mice from BC transfer (left) and AC transfer (right) following 48-hour *ex-vivo* stimulation with F85–93 peptide. Ad-SF40L was statistically different from all groups for both cytokines. Data shown is mean ± SEM representative of 2 independent experiments; n = 4 per group; *p < 0.05, **p < 0.01, ***p < 0.001 (one-way ANOVA with Bonferroni posttest).

We next set out to determine CD8 T cell functional activities in various immunization groups before and after RSV challenge. Figure 6A represents a schematic diagram of the adoptive transfer of CD8 T cells into naïve mice following immunization either before (BC) or after (AC) challenge. BC transfer from Ad-SF40L immunization led to an almost complete RSV clearance from the lungs of recipients, levels significantly lower than Ad-SF, RSV and FIRSV (p = 0.0165) that had no differences among them (Fig. 6B left). AC transfer, however, showed a spike in lung RSV titer when the CD8 T cells were isolated from FIRSV immunized donors whereas the other groups were not significantly different from the protection observed in BC transfer recipients (Fig. 6B right), revealing a significant deterioration of CD8 T cell function after challenge in the FIRSV group (see below for more discussion).

To analyze the effector response in the BC and AC transfer recipients, we measured the total RSV F-specific cytokine levels in the recipients. BC transfer resulted in significantly higher levels of IFN-γ (Fig. 6C left) and TNF-α (Fig. 6D left) in recipients of CD8 T cells from Ad-SF40L (p < 0.05) immunized donors compared to Ad-SF and FIRSV. IFN-γ expression considerably increased in Ad-SF40L AC transfer recipients while levels induced by Ad-SF, RSV and FIRSV immunization decreased or remained unchanged (Fig. 6C right). Importantly, IFN-γ and TNF-α were induced to barely detectable levels in AC transfer mice receiving CD8 T cells from FIRSV immunization (Fig. 6C,D right). Overall, Ad-SF40L gives rise to CD8 T cells that improve with RSV challenge as demonstrated by increased effector functions and TEMs (Fig. 4) whereas FIRSV induced CD8 T cells which are deficient in effector cytokine production, especially post RSV challenge, resulting in a substantial decrease of the capacity for viral clearance in CD8 T cell recipients.

### Ad-SF40L invokes long-lasting protection against RSV infection accompanied by a durable CD8 T cell effector memory response

**Figure 7:**
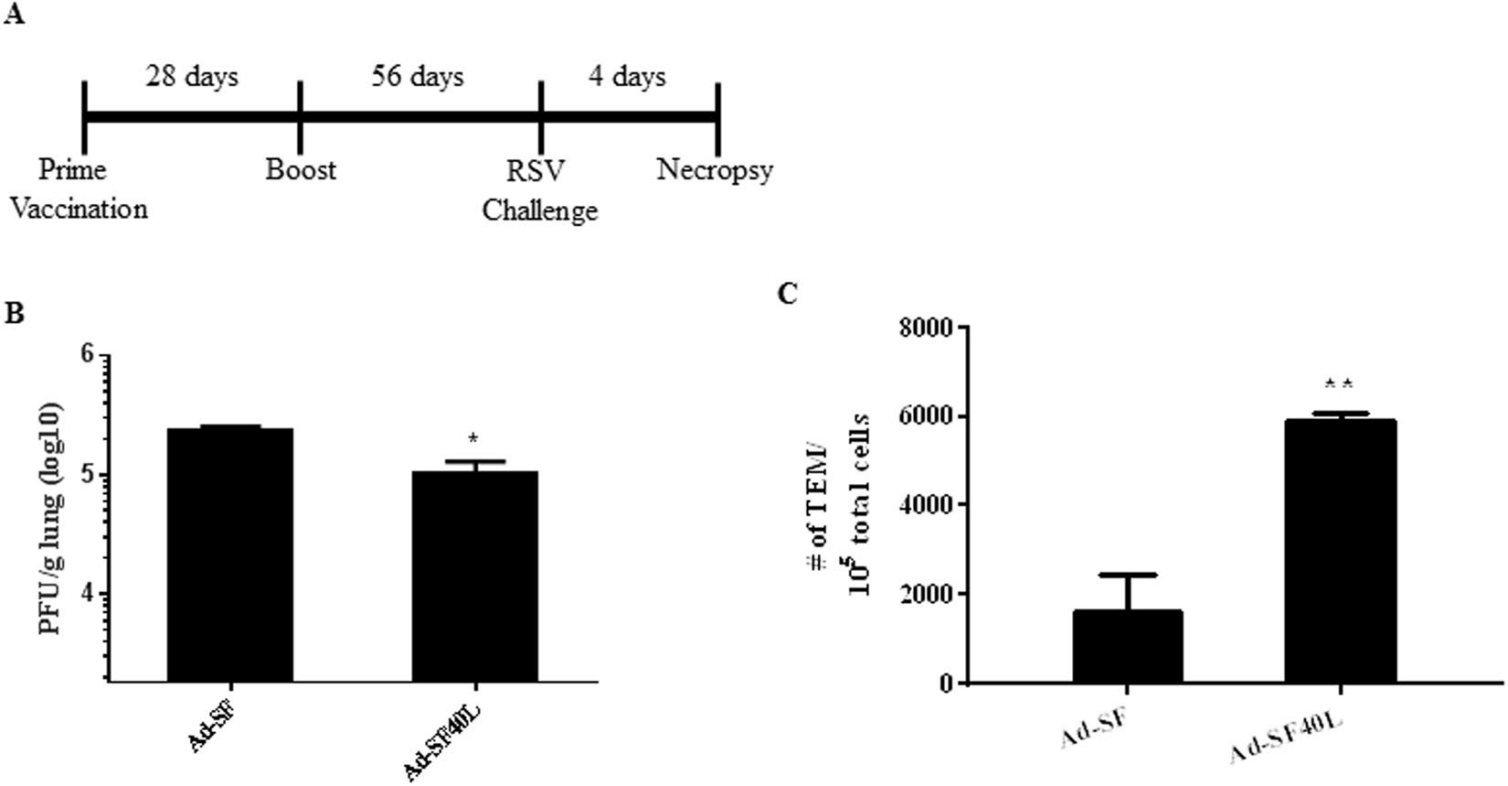
Ad-SF40L gives long-lasting protection from RSV infection accompanied by a durable CD8 T cell effector memory response. (**A**) Schematic diagram of the animal study with BALB/c mice. Mice were challenged 56 days after boost vaccination and necropsied 4 days after challenge. (**B**) Lung viral titer determined using plaque assay at necropsy. (**C**) Flow cytometry was used to determine the total number of TEMs (CD3+ CD8a+ CD44+ CD62L− CCR7−) in the spleen. Data shown is mean ± SEM; n = 4 per group; *p < 0.05, **p < 0.01 (unpaired Student’s t-test).

Finally, we determined if targeting CD40 could induce long-term protection. To this end, BALB/c mice were immunized with either Ad-SF or Ad-SF40L and challenged three months later with RSV (Fig. 7A). As shown in Fig. 7B, Ad-SF40L immunized mice were able to resolve the infection significantly better than Ad-SF (p = 0.0286). Importantly, significantly higher levels of TEM cells was observed in Ad-SF40L immunized mice (p = 0.0014) following challenge (Fig. 7C), which was comparable to the increase seen in short-term protection (Fig. 4B and Supplementary Fig. 3B). Taken together, Ad-SF40L effectively induces long-lasting protection in mice with a durable induction of TEMs.

## Discussion

Decades of effort have not resulted in a licensed vaccine for RSV. In addition to the ERD observed in the early clinical trials, results from most vaccines evaluated in recent clinical trials show a lack of efficacy^47–49^. Although a commercially-available monoclonal antibody against an epitope on the RSV F protein named palivizumab (Synagis®) could be administered to children with serious lower respiratory tract disease, it needs to be administered multiple times to reach therapeutic effects; in addition, this therapeutic antibody is costly and is also known to be ineffective in adults^50,51^. Clearly, vaccination is the most effective means of protecting children against RSV. Although there have been many recent studies exploring different modes of vaccination, antigen targets and animal models to gain further mechanistic insight^52–54^, there are important questions yet to be answered, particularly with respect to long-lasting immune responses and the role of memory CD8 T cells. The aim of this study was to investigate if enhancing immune response by using CD40 ligand as both an antigen-targeting molecule and immune modulator could result in robust and long-lasting CD8 T cell memory responses. This strategy of administering one protein expressing both the RSV antigen and CD40 targeting molecule is different from previous work reported in the literature. Specifically, Lee *et al*. showed enhanced viral clearance and decreased lung pathology in mice immunized with the RSV M2 protein along with a TLR3 agonist and anti-CD40 antibody^32^. Yet, while M2 is a potent antigen in inducing cell-mediated immune responses, it may not be as immunogenic as the RSV F protein^7^. In another study, Tripp *et al*. used live wild-type RSV as the vaccine with a separate intraperitoneal inoculation of mice with adenovirus-vectored CD40L and observed increased levels of Th1 cytokines and antigen-specific antibody production during a short-term observation period^33^. Another study involved a DNA vector expressing RSV F and/or G protein in conjunction with a separate DNA vector producing CD40L^34^. They also observed complete viral clearance but the antigen-specific antibodies were very low before the actual viral challenge took place; it is also unclear as to whether those antibodies have neutralizing activities. In our study, the neutralizing antibodies were markedly higher than non-CD40L immunization, suggesting that both neutralizing antibodies and CD8 T cells significantly contributed to protection, which is also supported by our observations that serum transfer from Ad-SF40L immunized mice did not afford protection as effectively as the donor mice themselves (Figs 2B and 5). Our findings also corroborate previous studies which revealed that TLR activation results in high avidity antigen-specific protective antibodies as the F protein employed in this study is a known TLR agonist^18^. Moreover, it is also known that TLR agonists and CD40L could synergistically facilitate B cell development^55^. Our findings together with these previous observations indicate that the selection of F protein as an antigen component in conjunction with CD40 stimulation could be a viable approach to maximize the protective potential of a candidate RSV vaccine.

Antibodies against the RSV F protein play an important role in the protection against RSV^18,56^. Analyses of human sera showed that majority of the RSV neutralizing antibodies target the F protein^57,58^. Structural analysis of antibodies raised from FIRSV immunization showed the exclusive induction of antibodies targeting poorly neutralizing epitopes, even though neutralizing epitopes were not hidden^56^. Moreover, low avidity of FIRSV-induced antibodies for protective epitopes on RSV has been attributed to the lack of protection. Indeed, lack of antibody affinity maturation due to poor toll-like receptor stimulation has been shown to result in non-protective antibodies^18^. Although the vital role of neutralizing antibodies in protection against RSV has been established, the role of non-neutralizing antibodies remains to be fully understood. In other viral infection such as influenza, non-neutralizing antibodies have been shown to promote antigen presentation to FcR+ cells, such as macrophages and/or lung phagocytes, leading to activation of CD8 T cells^59,60^. In our study, FIRSV induced low levels of neutralizing antibodies (Fig. 3C) but higher number of TEMs (Supplementary Fig. 3) compared to Ad-SF40L even though the number of FIRSV-induced TEM did not increase post challenge (Fig. 4B). Interestingly, while CD8 T cells contribute to viral clearance, they can also induce lethal immunopathology following RSV infection^22^. As such, a balance might be needed to ensure effective viral elimination without inducing ERD.

We then characterized the T cell-mediated immunity induced by Ad-SF40L to show the production of a strong effector memory CD8 T cell response upon RSV challenge. CD40L had a profound effect on RSV clearance and F-specific cytokine production in recipients after CD8 T cell transfer (Fig. 6). Ad-SF40L immunization elicited a CD8+ TEM population that increased in size upon encounter with the live virus indicating the presence of memory and antigen recognition capacity (Fig. 4B); importantly, the fold increase of TEMs due to RSV challenge remained comparable between short- and long-term protection in terms of memory CD8 T cell response (Fig. 7C), suggesting that the augmentation of CD8 T cell memory is long-lasting.

It is of note that following viral challenge the functional activities of CD8 T cells derived from FIRSV immunized animals significantly deteriorated (Fig. 6). Moreover, it is known that patients with severe lower respiratory tract disease may have insufficient cell-mediated immunity^61^. Clearly, the induction of memory CD8 T cells should be a vital element in evaluating the efficacy of RSV vaccines whereas the current RSV vaccine development like many other vaccines is focused on inducing a strong humoral response^62–64^.

It is worth mentioning that targeting CD40L more effectively eliminated virus and afforded long lasting protection, there is still limitation in our current work. Specifically, as the study presented in this report was mostly aimed at comparing the vectored vaccines delivered intranasally with FIRSV injected intramuscularly, we did not investigate the changes of resident CD8 T cells in the lung tissues, given intranasal delivery of alum-adjuvanted FIRSV was unsuccessful due to the viscosity of the vaccine preparation, while previous studies have shown that route of injection plays a crucial role in determining the immune responses at the site of infection^65^. Specifically, studies involving respiratory viruses have shown that intranasal administration results in robust pulmonary tissue-resident effector and memory CD8 T cells compared to intraperitoneal and intramuscular administration^65,66^. However, other studies have shown that both intranasal and intramuscular administration result in similar numbers of effector memory CD8 T cells in the spleen and lung vasculature^66^. Nevertheless, more experiments should be conducted to better decipher the roles of RSV-specific CD8 T cells in the lung tissues, given its crucial role in protection^65^, which is ongoing in our laboratories.

In summary, we present the first report on a fusion protein comprised of a RSV F antigen and CD40L, in which CD40L functions as both antigen-targeting molecular and immunomodulator. Our studies help better understand the mechanisms underlying CD8 T cell mediated short- and long-term protection against RSV infection and FIRSV-induced ERD with regards to CD8 T cell induction.

## Methods

### Generation of recombinant adenovirus

Constructs were designed to express the full RSV-A2 F protein (GenBank accession #KJ155694.1) as a secreted form with the inclusion of 23 amino acids from the human tyrosinase signal peptide (MLLAVLYCLLWSFQTSAGHFPRA; GenBank accession #AH003020) at the N-terminus (Ad-SF) as previously described^35^. A 27 amino acid fragment from the bacteriophage T4 fibritin trimerization motif (GYIPEAPRDGQAYVRKDGEWVLLSTFL) was added to the C-terminus of SF along with the complete mouse CD40L (GenBank accession #NM_011616) to form a trimeric, secreted protein, SF40L. Recombinant adenoviruses (Ad) were generated using the Directional TOPO® and the Gateway®-adapted ViraPower adenoviral expression vector system (Invitrogen) according to the manufacturer’s instructions.

Briefly, SF40L was synthesized by Bio S&T (Montreal, QC, Canada) in pBluescript II SK+. All PCR reactions were done using High Fidelity Platinum Pfx PCR kits (Life Technologies). Using the primers listed in Supplementary Table 1, SF40L and SF were isolated from pBluescript containing SF40L. The PCR products were then cloned into pENTR/SD/D-TOPO (Invitrogen) as per manufacturer’s instructions. Following transformation into *E*. *coli* and isolation of the plasmid, the sequence of the insert was confirmed. Next, a recombination reaction was done between the TOPO vector containing SF40L or SF and pAd/CMV/V5-DEST (Invitrogen). Once again, transformation, plasmid isolation and sequencing were conducted. Then, pAd-DEST vector was digested with *PacI* restriction enzyme to expose the viral inverted terminal repeats, phenol-chloroform extracted, ethanol-precipitated, and transfected into QBI-HEK 293 A cells. Following 80% cytopathic effect due to the production of adenoviruses, the cells and supernatant were harvested, lysed, and frozen at −80 °C. Purified stocks were made in QBI-HEK 293 A cells for animal studies by ultracentrifugation with a sucrose cushion. Adeno-X Rapid Titer Kit (Clontech Laboratories Inc.) was used for titration of the adenoviruses.

### Protein expression and immunofluorescence

HeLa cells were seeded at a density of 10,000 per well in growth media in a 96-well flat clear-bottom black plates and incubated overnight at 37 °C and 5% CO_2_. Next day, the cells were infected at a MOI of 100 with purified adenovirus. On the following day, infected cells were fixed with cold cytofix/cytoperm (BD Biosciences) for 10 min at 4 °C. After blocking with 3% IgG-free BSA diluted in wash buffer (1x PBS with 0.1% Tween 20) for 1 hour at 37 °C, the cells were stained with an unconjugated Rabbit RSV F monoclonal antibody (Sino Biological; clone #009) for 1 hour at 37 °C. Then, a mixture of Alexa Fluor 594-conjugated anti-rabbit IgG (Abcam) and FITC-conjugated anti-mouse CD40L (Invitrogen) was added for 1 hour at 37 °C. The cells were imaged using the EVOS FL microscope: Alexa Fluor 594 in the RFP and FITC in the GFP channel.

### Cells, viruses and vaccines

HEp-2 (ATCC: CCL-23) and HeLa cells (ATCC: CCL-2) were grown in Dulbecco’s Modified Eagle Medium (DMEM) supplemented with 1.5 g/l sodium bicarbonate, 2 mM Glutamax, 1 mM HEPES, 20 U/ml Penicillin, 0.02 mg/ml Streptomycin, and 10% FBS. Finally, QBI-HEK 293 A were cultured in DMEM with 1.5 g/l sodium bicarbonate, 25 mM HEPES, 20 U/ml Penicillin, 0.02 mg/ml Streptomycin, and 10% FBS.

RSV-A2 (ATCC: VR-1540) was grown in HEp-2 cells according to supplier’s instructions and sucrose-purified for animal studies. FIRSV was prepared with the RSV-A2 strain in HEp-2 cells as described elsewhere^17^.

### Animal studies

Six-week old female BALB/c mice (Charles River, Saint Constant, QC) were used for all animal studies. Ad-SF and Ad-SF40L were administered intranasally at 10^8^ PFU per mouse, RSV at 10^5^ PFU intranasally and FIRSV at 10^6^ PFU intramuscularly. All mice were challenged intranasally with 5 × 10^5^ PFU of RSV-A2. Intranasal inoculations were given in 25 µl per mouse and intramuscular in 50 µl. Each immunization was administered twice at the same dose and route 28 days apart. Fourteen days after the second immunization, the mice were challenged and 4 days post challenge euthanized for blood and tissue collection. All animal experiments were reviewed and approved by Institutional Animal Care and Use Committee of Health Canada and were conducted in accordance with Institutional Animal Care and Use Committee of Health Canada guidelines and regulations.

### Lung viral titer

Lungs were removed four days post RSV challenge and tittered as described elsewhere^17^. Briefly, lungs were collected in serum free RPMI media and weighed prior to mechanical homogenization. The homogenates were clarified using centrifugation and the supernatants were frozen at −80 °C until further use. Serial dilutions of the supernatant were done and incubated on HEp-2 cells for 2 hours at 37 °C. A 1:1 overlay of 2x DMEM media and 0.8% agarose was added. Following 6 days of incubation, the overlay was removed and the cell monolayer was stained with crystal violet before counting plaques. Results are expressed as PFU/g lung tissue.

### Histopathology

Four days post RSV challenge, whole lungs were collected from the BALB/c mice and fixed in 10% neutral buffered formalin. They were then trimmed, processed and embedded into paraffin blocks. Five-micron Hematoxylin and Eosin (H&E) and Periodic Acid Schiff (PAS) stained slides were made for evaluation. Scoring was done by a veterinary pathologist who was blinded to the experimental design. The lesion assessment protocol outlined by Weiss *et al*.^67^ was adopted. Perivascular leukocytic infiltration was evaluated where 1 means within normal parameters; 2 means small numbers of solitary cells with uncommon aggregates; 3 means multifocal small to moderate aggregates; 4 means moderate to high cellularity with multifocal large cellular aggregates that may be expansive into adjacent tissues. Mucus was visualized with PAS stain and graded as follows: 1 means none; 2 means epithelial mucinous hyperplasia with none to rare luminal mucus accumulation in airways; 3 means epithelial mucinous hyperplasia with luminal mucus accumulation in airways; 4 means there is severe mucinous hyperplasia with airway obstruction by mucus. Total pathological score was calculated as the average of the individual scores.

### ELISA

Serum from immunized and challenged mice was collected for determination of IgG, IgG1 and IgG2a titer. Ninety six-well plates were coated with recombinant RSV F protein (Sino Biological) overnight at 4 °C. Next day, the plates were washed and blocked with BSA in PBS containing 0.05% Tween 20 for 2 hours at 37 °C. Serial dilutions of the mouse serum in blocking buffer were then added for 1 hour at 37 °C. After washing, HRP-conjugated anti-mouse IgG (GE Healthcare Life Sciences), anti-mouse IgG1 or IgG2a (Jackson Immunoresearch Laboratories) were added for 1 hour at 37 °C. The plates were again washed and Tetramethylbenzidine substrate (Cell Signaling Technology) was added for 20 min at room temperature. The reaction was then stopped with 0.16 M sulfuric acid. The plates were read spectrophotometrically at 450 nm.

### Microneutralization

RSV-neutralizing ability of the serum from immunized and challenged mice was determined. Serial dilutions of the serum were incubated with 800 PFU of purified RSV-A2 for 1 hour at 37 °C, 5% CO_2_. The virus-antibody mixture was added to HEp-2 cells seeded the previous day and incubated at 37 °C. After 3 days, the cells were fixed with ice-cold methanol for 10 min at room temperature, air-dried, and blocked with 5% non-fat dry milk in PBS containing 0.1% Tween 20 for 2 hours at 37 °C. Then, the plates were washed and a HRP-conjugated anti-RSV (Meridian Life Science) was added for 1 hour at 37 °C. The plates were again washed and Tetramethylbenzidine substrate (Cell Signaling Technology) was added for 20 min at room temperature. The reaction was then stopped with 0.16 M sulfuric acid. The plates were read spectrophotometrically at 450 nm.

### Flow cytometry for surface and intracellular markers

Cells from spleens were isolated from immunized mice before or 4 days after challenge. Single-cell suspensions were washed with PBS and first, stained with Fixable Viability Dye eFluor® 506 (eBioscience) for 30 min at 4 °C, then, with purified anti-mouse CD16/CD32 (eBioscience) as a Fc block for 5 min. Next, cells were washed with FACS wash buffer (PBS with 1% BSA and 0.05% sodium azide) and stained with PE-CF594-conjugated anti-mouse CD40 (clone 3/23) or a panel with BV786-conjugated anti-mouse CD3 (clone 145-2C11), FITC-conjugated anti-mouse CD8a (clone 53–6.7), BV421-conjugated anti-mouse CD44 (clone IM7), PE-Cy7-conjugated anti-mouse CD62L (clone MEL-14), and AF647-conjugated anti-mouse CD197 (clone 4B12). All antibodies were purchased from BD Biosciences. Stained samples were fixed with 1% paraformaldehyde.

For intracellular staining, splenocytes were stimulated with an immunodominant H-2K^d^-restricted RSV F_85–93_ peptide (KYKNAVTEL; ProImmune) at 5 µg/ml, along with GolgiPlug (BD Biosciences) for 4 hours at 37 °C, 5% CO_2_. Stimulated cells were washed and stained sequentially with the viability dye, Fc block, FITC-conjugated anti-mouse CD8a (clone 53–6.7), and incubated with BD cytofix/cytoperm (BD Biosciences) for 20 min. The permeabilized cells were then washed with 1x perm wash (BD Biosciences) and stained with PE-conjugated anti-mouse TNF-α (clone MP6-XT22) for 30 min. For both surface and intracellular stained samples, using a BD LSRFortessa flow cytometer, 100,000 viable singlet events for spleen and 50,000 viable singlet events for lymph node were recorded. FMO controls and compensation beads were used where appropriate to correct for spectral overlap. Data analysis was completed using FACSDiva version 8.0.1.

### Secreted cytokines

Splenocytes were stimulated with an immunodominant H-2K^d^-restricted RSV F_85–93_ peptide (KYKNAVTEL; ProImmune) at 5 µg/ml for 48 hours at 37 °C, 5% CO_2_. Supernatants were collected following centrifugation and stored at −80 °C for later analysis. A ProcartaPlex Mouse Cytokine Panel (eBiosciences) was used to determine the levels of IFN-γ and TNF-α in the supernatants. The samples were read on a Luminex 200 System (xMAP Technology). Data analysis was performed using MILLIPLEX Analyst version 5.1 for determining the pg/ml of each cytokine.

### Passive Serum Transfer

Serum was aseptically collected and pooled from 25 immunized BALB/c mice 4 days after challenge. Naïve 6-week-old BALB/c mice were intraperitoneally injected 4 times with 300 µl of donor serum 3 days, 2 days, 1 day before RSV challenge and on the day of the challenge^35^. Four days post-challenge, mice were euthanized for tissue collection (Fig. 5A).

### Adoptive T cell transfer

Spleens were aseptically extracted from 25 immunized BALB/c mice before or after RSV challenge. Splenocytes were isolated and pooled for CD8 and CD4 T cells extraction. Dynabeads® Untouched Mouse CD8 Cells Kit and CD4 Cells Kit (Life Technologies) were used according to manufacturer’s instructions. Prior to transfer, flow cytometry staining was done to determine purity of the resulting CD8 and CD4 T cells using PerCP-conjugated anti-mouse CD8a (clone 53–6.7; BD Biosciences), and FITC- conjugated anti-Mouse CD4 (clone RM4-4; eBiosciences). On the same day, 4.6 million CD8 or CD4 T cells at 80% and 87% purity, respectively, were injected intravenously, via the tail vein, into naïve 6-week-old BALB/c mice^35^. Three days later, mice were challenged intranasally with RSV and euthanized 4 days post-challenge (Fig. 6A and Supplementary Fig. 4A).

### Statistical analysis

Analysis was conducted using unpaired Student’s t-test, one-way or two-way ANOVA where appropriate. Bonferroni posttest was used to adjust for multiple comparisons between different test groups. Tests were done at a 5% significance level. All statistical analyses were performed using GraphPad Prism 6 software.

## Electronic supplementary material

Supplementary Data

## Data Availability

The datasets generated during and/or analyzed during the current study are available from the corresponding author on reasonable request.
