# Supplementary material for: Targeting CD40 enhances antibody- and CD8-mediated protection against respiratory syncytial virus infection
Source: Sci Rep. 2018 Nov 9;8:16648. doi: 10.1038/s41598-018-34999-z (PMC6226510; doi:10.1038/s41598-018-34999-z)
Supplement: Supplementary file 1 — Supplementary Data [file 41598_2018_34999_MOESM1_ESM.pdf]

Supplementary Information

Targeting CD40 enhances antibody- and CD8-mediated protection against respiratory syncytial virus infection

Abenaya Muralidharan[1,3], Marsha Russell[1], Louise Larocque[1], Caroline Gravel[1], Changgui Li[2], Wangxue Chen[4], Terry Cyr[1], Jessie R. Lavoie[1], Aaron Farnsworth[1], Michael Rosu-Myles[1,3], Lisheng Wang[3], Xuguang Li[1,3,*]

[1]Centre for Biologics Evaluation, Biologics and Genetic Therapies Directorate, HPFB, Health Canada and WHO Collaborating Center for Standardization and Evaluation of Biologicals, Ottawa, ON, Canada.

[2]National Institute for Food and Drug Control and WHO Collaborating Center for Standardization and Evaluation of Biologicals, Beijing, China

[3]Department of Biochemistry, Microbiology and Immunology, Faculty of Medicine, University of Ottawa, Ottawa, ON, Canada

[4]Human Therapeutics Portfolio, National Research Council of Canada, Ottawa, ON, Canada

[*]Corresponding author

Supplementary Table 1: Primers used for cloning and sequencing of Ad-SF and Ad-SF40L constructs.

Name	Forward Primer (5' – 3')	Reverse Primer (5' – 3')
**Primers for Cloning:**		
Ad-SF40L	CACCGCCGCCACCATGCT	TCACAATTTCAACAAGCCAAA
Ad-SF	CACCGCCGCCACCATGCT	TCAATTGCTAAAGGCAATATTATTAATGCCGCTC
**Primers for sequencing:**		
SF40L-1F	CGCCGCCACCATGCTGCT	
SF40L-2F	AAAGGTAAAGCTCATCAAGCAGGAGC	
SF40L-3F	CAACAAGGCAGTAGTTTCCC	
SF40L-4F	GAGCAACAACGTCCAGATCGTGA	
SF40L-5F	TCTGCCTTCTGAAGTCAACTTGTGCA	
SF40L-6F	TATTAATTTCTATGACCCCCTGG	
SF40L-7F	TGGCTATATACCTGAGGCCCCCA	
SF40L-8F	CAAAGGCGAGGGGTCTCTGT	
SF40L-9F	GGTGACATTTTGCTCAAATCGCG	

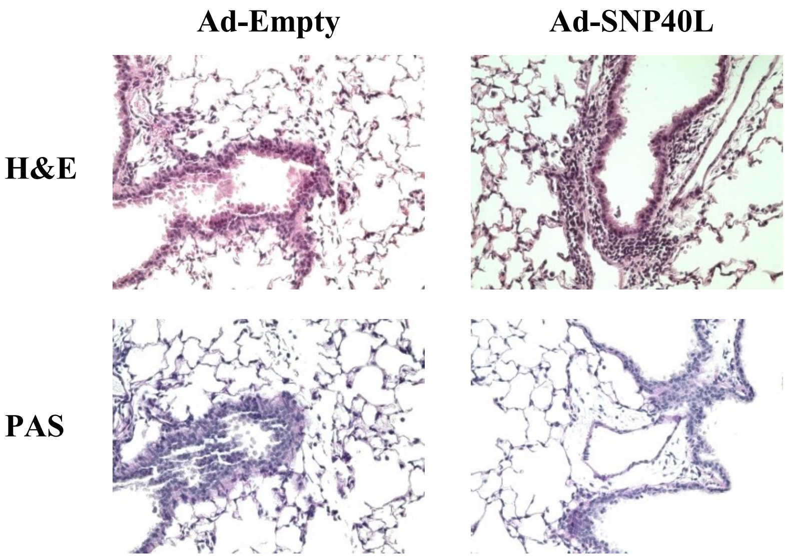

Supplementary Fig. 1: Representative image of H&E and PAS stained lungs of mice post-challenge. BALB/c mice were intranasally immunized twice with control vaccines, Ad-Empty or Ad-SNP40L, to determine the antigen specificity of the observed protection. Lungs were H&E and PAS stained 4 days post challenge for perivascular leukocyte infiltrate and mucus scoring, respectively. Representative images at 40x magnification are shown.

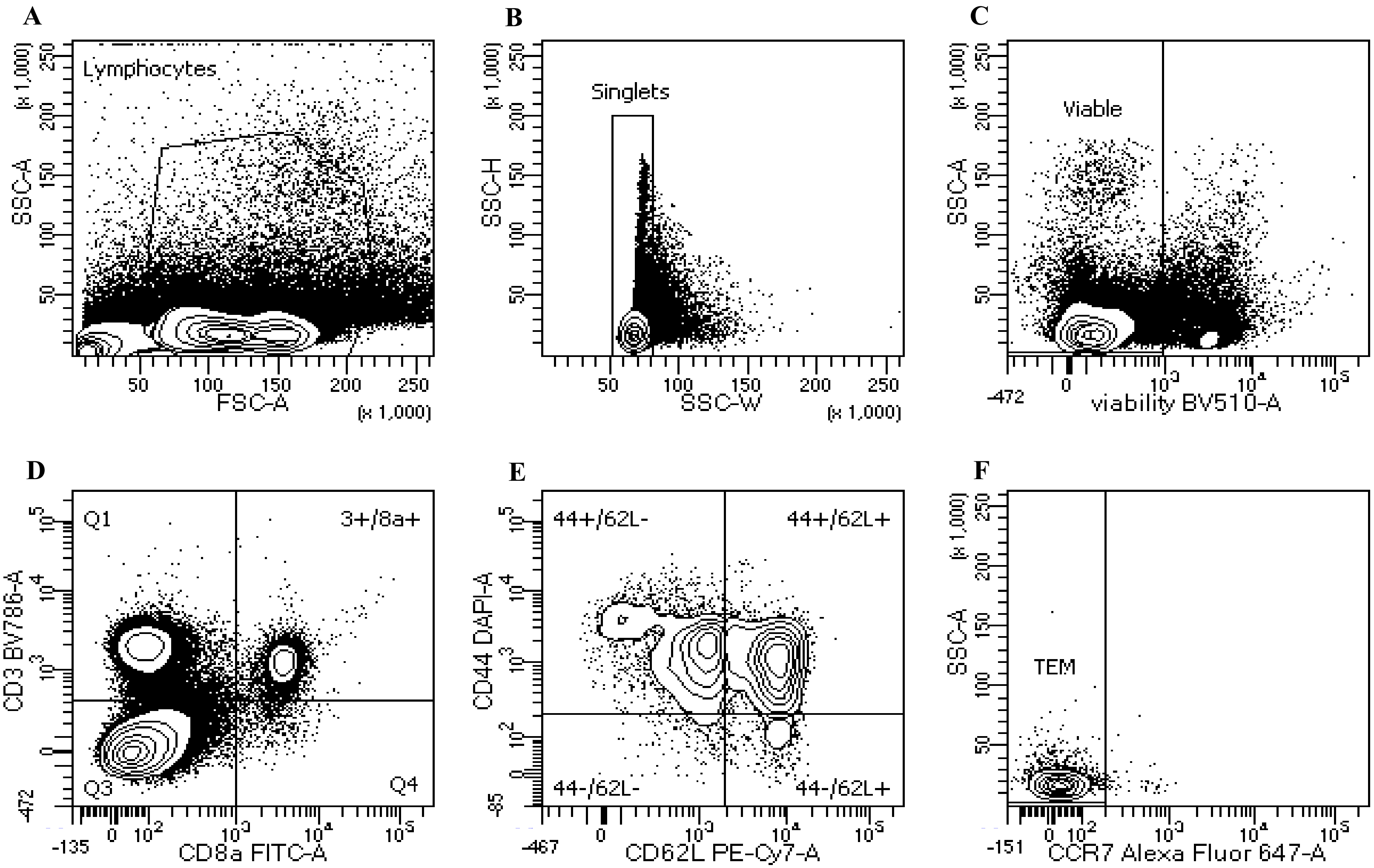

Supplementary Fig. 2: TEM gating strategy. Splenocytes were isolated from immunized BALB/c mice before or after challenge and stained for flow cytometry analysis. A FSC/SSC plot was done to gate for lymphocytes (**A**). Singlets were selected from the lymphocytes (**B**) and further gated for viable cells (**C**). The viable cells were then gated for CD3+CD8α+ (**D**). Next, the CD3+CD8α+ cells were analyzed for CD44+CD62L- (**E**), which were finally gated for CCR7- population (**F**). This CD3+ CD8a+ CD44+ CD62L- CCR7- is denoted as effector/effector memory CD8 T cells or TEM.

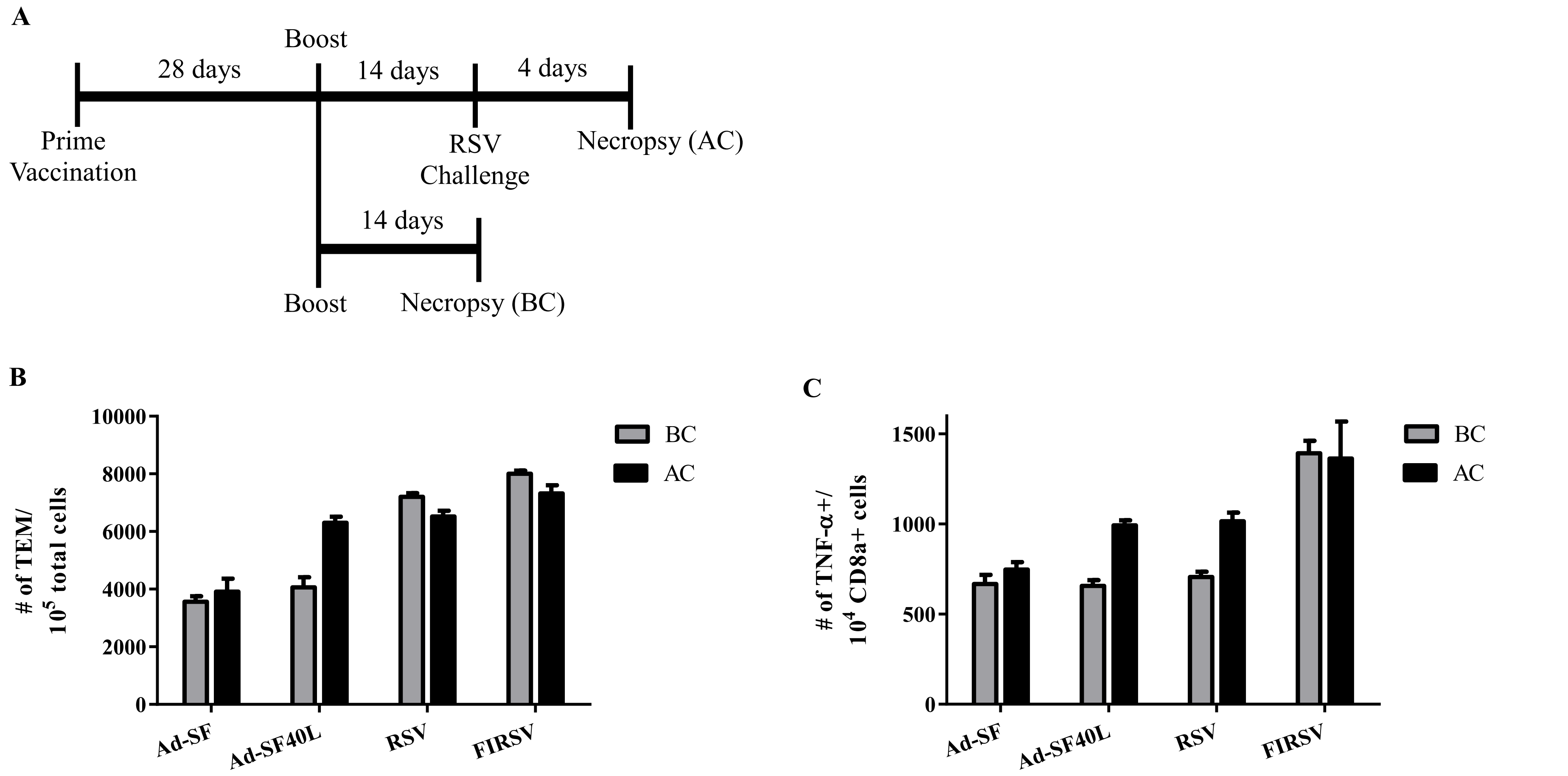

Supplementary Fig. 3: Increase in CD8 T cell effector phenotype and function following challenge is unique to Ad-SF40L immunization. (**A**) Schematic diagram of the animal study with BALB/c mice. Mice were necropsied for tissue collection before or after challenge. (**B**) Flow cytometry was used to determine the TEM population (CD3+ CD8a+ CD44+ CD62L- CCR7-) in the spleen. (**C**) Intracellular cytokine staining was done following 4-hour ex-vivo stimulation with F85-93 peptide to analyze the number of CD8α+ TNF-α+ cells in the spleen using flow cytometry. Data shown is mean ± SEM; n = 4 per group representative of 2 separate experiments; $^{*}p < 0.05$, $^{**}p<0.01$ (one-way ANOVA with Bonferroni posttest).

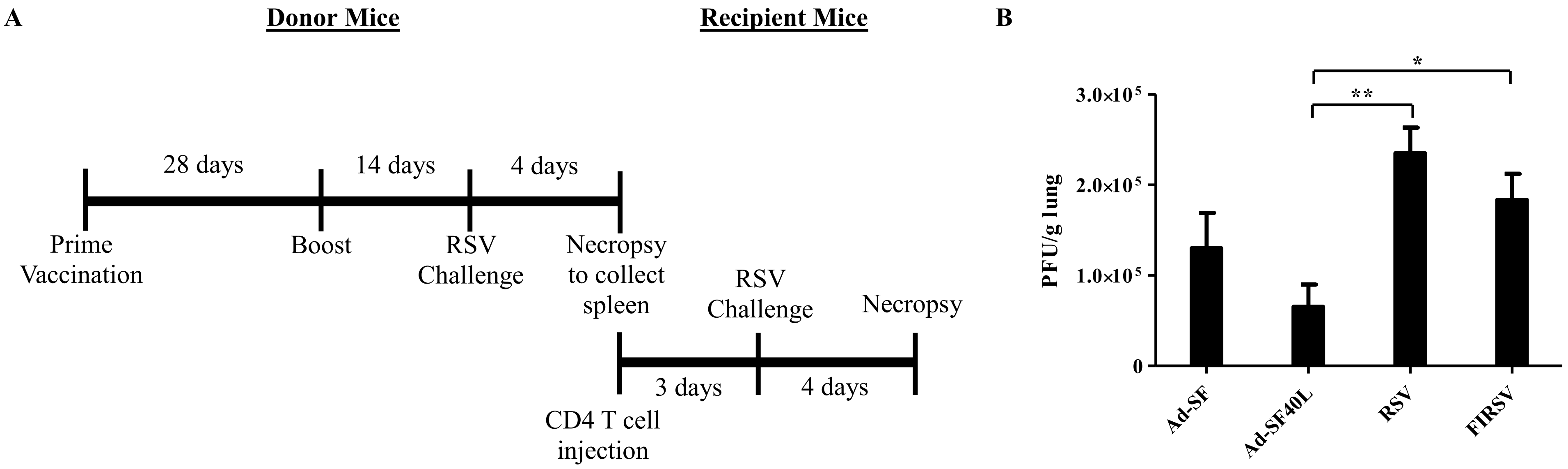

Supplementary Fig. 4: CD40L does not enhance CD4 T cell-induced protection. (**A**) Schematic diagram of the animal study timeline. Adoptive CD4 T cell transfer was done from immunized and challenged donor mice into naïve recipient mice. CD4 T cells were isolated from spleens of donor mice using a magnetic bead kit and checked for purity prior to transfer. (**B**) Four days post-challenge, lungs from the recipient mice were collected for viral titer determination using plaque assay. There were no significant differences between Ad-SF and Ad-SF40L group. Data shown is mean ± SEM; n = 4 per group; *$p < 0.05$, **$p<0.01$ (one-way ANOVA with Bonferroni posttest).
